# Cadmium and Plant Development: An Agony from Seed to Seed

**DOI:** 10.3390/ijms20163971

**Published:** 2019-08-15

**Authors:** Michiel Huybrechts, Ann Cuypers, Jana Deckers, Verena Iven, Stéphanie Vandionant, Marijke Jozefczak, Sophie Hendrix

**Affiliations:** Environmental Biology, Centre for Environmental Sciences, Hasselt University, B-3590 Diepenbeek, Belgium

**Keywords:** cadmium, oxidative stress, cell cycle, cell wall, germination, reproduction, plant growth and development

## Abstract

Anthropogenic pollution of agricultural soils with cadmium (Cd) should receive adequate attention as Cd accumulation in crops endangers human health. When Cd is present in the soil, plants are exposed to it throughout their entire life cycle. As it is a non-essential element, no specific Cd uptake mechanisms are present. Therefore, Cd enters the plant through transporters for essential elements and consequently disturbs plant growth and development. In this review, we will focus on the effects of Cd on the most important events of a plant’s life cycle covering seed germination, the vegetative phase and the reproduction phase. Within the vegetative phase, the disturbance of the cell cycle by Cd is highlighted with special emphasis on endoreduplication, DNA damage and its relation to cell death. Furthermore, we will discuss the cell wall as an important structure in retaining Cd and the ability of plants to actively modify the cell wall to increase Cd tolerance. As Cd is known to affect concentrations of reactive oxygen species (ROS) and phytohormones, special emphasis is put on the involvement of these compounds in plant developmental processes. Lastly, possible future research areas are put forward and a general conclusion is drawn, revealing that Cd is agonizing for all stages of plant development.

## 1. Introduction

Cadmium (Cd) pollution, as a consequence of both geological and anthropogenic activities, affects many regions worldwide [1]. Although Cd is non-essential and no specific Cd uptake mechanisms have been identified in plants, it is taken up in root cells through transporters for essential bivalent cations such as calcium (Ca), iron (Fe), manganese (Mn) and zinc (Zn) [2]. Depending on the plant species and growth conditions, plants can endure low Cd concentrations, but in general Cd disturbs photosynthesis, respiration and the uptake of water and nutrients. As a consequence, Cd pollution negatively affects plant growth and development, thereby significantly reducing crop yield [3]. 

This Cd-induced phytotoxicity is related to its ability to bind to thiol, histidyl and carboxyl groups of structural proteins and enzymes, thereby interfering with their function. Furthermore, Cd can also disturb protein function by replacing essential ions in their active sites due to its strong chemical similarity with other divalent cations. Despite its non-redox-active nature, Cd indirectly induces the production of reactive oxygen species (ROS), resulting in an oxidative challenge, which is defined as an imbalance between cellular pro- and antioxidants in favor of the former [4,5]. This Cd-induced ROS production is achieved through multiple mechanisms. Firstly, Cd is able to replace Fe in various proteins, thereby increasing free cellular Fe levels. As a redox-active metal, Fe can directly induce ROS production through Fenton and Haber-Weiss reactions [6]. Furthermore, Cd indirectly induces ROS production by depleting cellular levels of the non-enzymatic antioxidant glutathione (GSH), as a consequence of increased phytochelatin (PC) synthesis. The latter contributes to Cd chelation but reduces the amount of GSH available for antioxidative defense [7,8]. In addition, indirect Cd-induced ROS production can result from its ability to inhibit enzymes involved in antioxidative defense mechanisms [8]. Cadmium can also contribute to ROS production through its effects on ROS-producing enzymes such as nicotinamide adenine dinucleotide phosphate (NADPH) oxidases and increases ROS production in plant organelles by interfering with metabolic processes such as photosynthesis, respiration and photorespiration [4].

When present in elevated concentrations, ROS can evoke damage to a multitude of cellular macromolecules including lipids, proteins and DNA. However, ROS are not only damaging agents, but are also key players in signal transduction during physiological processes as well as responses to biotic and abiotic stresses [9,10,11]. In order to enable ROS-induced signaling and prevent damage to cellular macromolecules, ROS levels should be tightly controlled. To this end, plants have developed an extensive antioxidative defense system consisting of both enzymatic antioxidants such as superoxide dismutase (SOD), catalase (CAT) and several peroxidases and non-enzymatic antioxidants such as the water-soluble GSH and ascorbate (AsA) and the lipid-soluble carotenes and α-tocopherol [9]. 

Besides interfering with ROS homeostasis, Cd exposure also affects phytohormone signaling [12,13]. It was shown, for example, that Cd exposure induces a fast and transient increase in ethylene levels in *Arabidopsis thaliana* through increasing the expression of 1-aminocyclopropane-1-carboxylate (ACC) synthase 2 (*ACS2*) and *ACS6*, which are involved in the biosynthesis of the ethylene precursor ACC [14]. Furthermore, Cd was shown to enhance ethylene and jasmonic acid (JA) concentrations in *Pisum sativum* [15] and abscisic acid (ABA), salicylic acid (SA) and JA levels in *Oryza sativa* [16]. It also interfered with auxin (AUX) homeostasis in *A. thaliana* seedlings, where it significantly altered AUX concentrations and distribution in primary root tips and cotyledons. It decreased indole-3-acetic acid (IAA) content, enhanced IAA oxidase activity and affected transcript levels of putative AUX biosynthetic and catabolic genes [17]. Similarly, a negative effect of Cd exposure on AUX biosynthesis, transport and distribution was also reported in *O. sativa* [18]. Interestingly, extensive cross-talk exists between ROS and phytohormones in plant development and stress tolerance, as was recently reviewed by Xia and colleagues (2015) [19]. 

In order to improve plant growth on Cd-polluted soils, for example for phytoremediation purposes [20], it is of crucial importance to increase our knowledge on the mechanisms underlying the negative impact of Cd exposure on plant development. Therefore, the main aim of this work is to provide an overview of Cd-induced effects on plant growth and development, specifically addressing (1) seed dormancy and germination, (2) vegetative plant growth and (3) reproductive plant growth. Important underlying mechanisms are discussed, highlighting the involvement of ROS and phytohormones. Furthermore, perspectives for future research are proposed, focusing on the investigation of transgenerational effects of Cd exposure and the involvement of the plant microbiome in response to Cd stress.

## 2. Seed Germination

Seed germination, accompanied with a release from seed dormancy, is one of the most important events in a plant’s life cycle. It determines whether a plant is willing to take the risk of environmental exposure in order to reach reproductive maturity and produce seeds of its own. In the past, this trait has been exploited in cereals to obtain seeds with a low dormancy level, so that when the seeds were sown onto the field, they would germinate quickly and evenly. This led to the problem known as pre-harvest sprouting, during which seeds already germinate when they are still attached to the mother plant, resulting in a significant reduction of yield and quality of the seeds [21,22,23]. 

Abscisic acid and gibberellic acid (GA) are the major phytohormones regulating germination. They act antagonistically, with high levels of ABA causing the preservation of a dormant seed and high levels of GA initiating germination [24,25]. The ABA/GA ratio thereby acts as a central hub integrating environmental signals [26]. However, recent studies also indicate a role for other plant hormones. Auxin, previously thought not to have an important role in seed germination, acts alongside ABA in keeping dormancy high [27]. Furthermore, AUX and ABA are dependent on each other in this process, as AUX operates by keeping the expression of a major ABA signaling downstream regulator, *ABSCISIC ACID INSENSITIVE 3* (*ABI3*), high [28]. In addition, ABA represses the elongation of the embryonic axis through AUX-regulated signaling [29]. Another hormone, ethylene, works in the opposite way. Ethylene is able to stop the inhibitory effect of ABA on endosperm weakening, thereby facilitating seed germination [30,31]. Several other phytohormones including brassinosteroids, cytokinins (CK), JA, strigolactones and SA, have been shown to either stimulate or inhibit germination. However, their mutual interactions and importance in this process are still unclear [27]. 

Next to the hormonal regulation of seed germination, the event is also characterized by an increase in ROS levels, especially hydrogen peroxide (H_2_O_2_) [32]. Reactive oxygen species are assumed to function upstream of the hormonal interactions by stimulating GA biosynthesis and metabolism and inducing ABA catabolism [33,34]. However, Bahin and colleagues (2011) stated that ROS mainly function through GA signaling since H_2_O_2_ did not influence ABA metabolism and signaling in *Hordeum vulgare* seeds [35]. On the other hand, ABA was shown to prevent the accumulation of ROS within the embryonic axis of the seeds [33]. Currently, the interactions between ROS and ABA/GA are still under debate [36]. The role of ROS in seed germination is dual and is defined as the oxidative window of germination [37]. Too low concentrations of ROS within the seed will fail to induce seed germination, whereas excessive ROS levels will lead to irreversible seed damage. In dry seeds, ROS levels tend to be rather low [37]. This is accomplished by maintaining a high level of antioxidant capacity scavenging ROS molecules. Depletion of antioxidants leads to a ROS increase, which initiates germination under favorable conditions [38]. Once H_2_O_2_ is elevated, it has the capacity to selectively oxidize mRNAs and proteins [39]. In *A. thaliana* seeds, ROS-induced carboxylation of 12S cruciferins, the major storage proteins, occurs. Upon seed imbibition, these oxidized molecules disappear rapidly, indicating their role in early seedling establishment [40]. A similar mechanism of proteome oxidation was found in sunflower seeds [41]. Furthermore, upon exposure to methylviologen, a ROS-inducing agent, genes involved in Ca and redox signaling were differentially expressed [33]. Hou and colleagues (2019) found that three major events were important in releasing *Leymus chinensis* seeds from dormancy [42]. A decrease in proteins related to AsA and aldarate metabolism accompanied with an increase of thioredoxins (Trx) changed the antioxidant system and led to an increase in ROS. This was followed by the oxidation of stored mRNAs and proteins. Furthermore, an increase in β-tubulin led to cytoskeleton changes and resulted in physical dormancy release by transporting substances related to germination and cell wall loosening. Thirdly, these cytoskeleton changes in turn affected chromatin remodeling and proteins [42]. 

Surrounded by a rigid seed coat, the plant embryo is well protected from environmental stresses [43,44]. Germination is initiated with the uptake of water followed by embryonic expansion [45]. Metabolic reactivation accompanied with metabolic respiration is characterized by a steep increase in oxygen consumption just after imbibition of the seed. Furthermore, protein synthesis, DNA repair and remobilization of stored reserves are essential processes in the successful germination of the seeds [46]. Physical constraints are imposed by outer seed tissue, and seed germination involves rupture of the testa and the endosperm [47]. Upon imbibition of the seed, the testa becomes more permeable over time and Cd content begins to increase in inner seed tissues [48]. Thereby, genotypic variations of seed coat permeability might be an important factor contributing to the effects of metals on seed germination [49,50]. Subsequently, Cd-inhibited seed germination occurs in a dose-dependent manner [51,52,53]. Seeds of *Trigonella foenum-graecum* exposed to solutions of chromium (Cr), lead (Pb) and Cd showed that Cd had the strongest germination inhibition effect at 10 mg L^−1^, which was the highest concentration tested [54]. Likewise, in *Triticum aestivum*, less Cd than Pb was needed to inhibit the seed germination process [55]. Nevertheless, large differences can occur between plant species. Ahsan and colleagues (2007) reported that adding 1 mM of CdCl_2_ to the solution completely inhibited seed germination of the *O. sativa* cultivar Hwayeong [56]. Seeds of *H. vulgare* were apparently more tolerant to Cd and their germination rate was only fully inhibited around 9.5 mM CdCl_2_ [57]. In addition, Cd tolerance of germination might differ largely within one plant species, as was shown within both *T. aestivum* and *O. sativa* cultivars [49,51]. Some cultivars still germinate vigorously with high Cd concentrations, while others will fail. Interestingly, under the threshold of 0.5 mM CdCl_2_, most tested *O. sativa* cultivars showed an increased germination compared to the control seeds [49]. A similar observation was made by Lefèvre and colleagues (2009) with *Dorycnium pentaphyllum* Scop. seeds [58]. Here, adding 10 µM CdCl_2_ also significantly increased germination compared to control seeds, while after exposure to 1 mM CdCl_2_ for 17 days, no more than 40% germination was obtained. These last findings could be indicative for seed germination stimulation at low Cd concentrations, i.e., hormesis, which are undoubtedly dependent on the plant species examined. 

**Figure 1:**
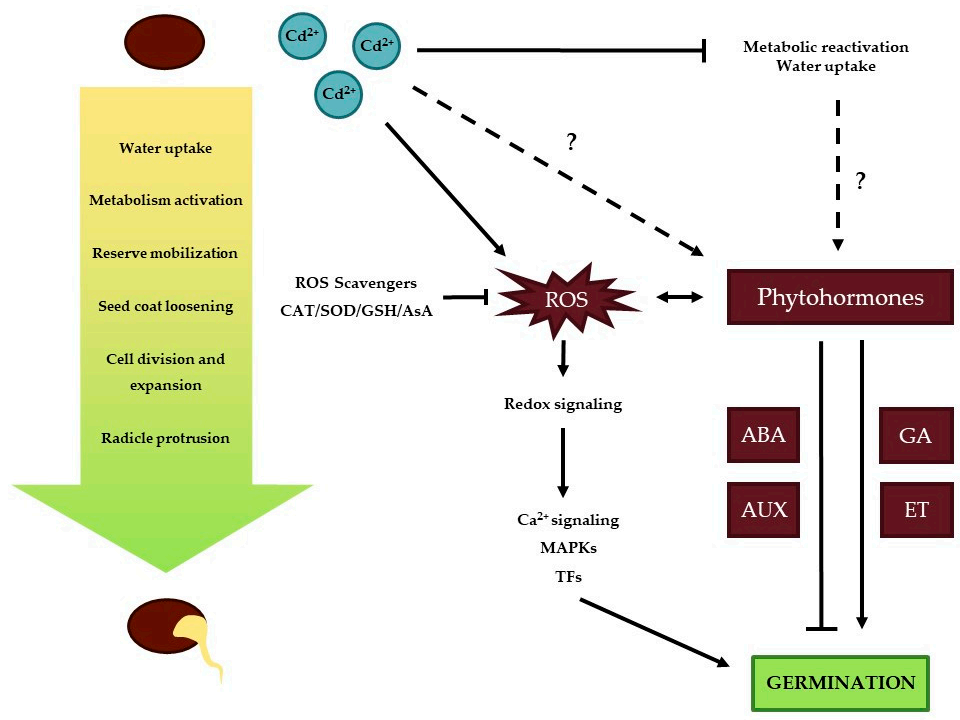
Possible interference mechanisms of cadmium on the process of seed germination. Cadmium (Cd) negatively affects metabolic reactivation by reducing levels of hydrolyzing enzymes, starch mobilization and seed imbibition. Furthermore, it can alter redox signaling via calcium (Ca), mitogen-activated protein kinases (MAPKs) and transcription factors (TFs) and the level of phytohormones such as abscisic acid (ABA), auxin (AUX), giberrellic acid (GA) and ethylene (ET). Both are of major importance in the seed germination process. One-way arrows: indicate a stimulating effect, whereas T-shaped arrows represent an inhibitory effect. Two-way arrows signify an interaction and dashed lines indicate effects which are still uncertain.

Cadmium is known to inhibit seed germination through different mechanisms (Figure 1). In *Vigna unguiculata* seeds, the inhibitory effect of Cd was proposed to be due to an impairment of water uptake, thereby limiting the water availability for the developing embryo [59]. A limited water supply is not the only problem for proper germination. An inhibition of starch mobilization from the endosperm accompanied with an impaired translocation of soluble sugars to the embryonic axis can lead to further starvation of this embryonic axis [50]. A reduction of hydrolyzing enzymes, such as α-amylase, proteases and acid phosphatases, in *Sorghum bicolor* seeds was suggested to be responsible for this reduced storage mobilization. A decrease in α-amylase activity has been reported multiple times in relation to a decrease in starch release from cotyledons [50,60,61]. He and colleagues (2008) pointed out that Ca is a vital element for amylase activity and the replacement of the chemically similar Cd ion could disrupt normal enzyme functioning [62]. Furthermore, in radish seeds, a direct competition for Ca-calmodulin binding sites occurred between Ca and Cd ions [63]. The interaction between Ca and calmodulin is suggested to serve a role in metabolic activation during the early phases of seed germination [64]. An alteration in the remobilization process is also observed in *Vicia faba* seeds by the leakage of soluble sugars and amino acids into the imbibition medium, which is probably related to the loss of membrane integrity [65]. An increase of malondialdehyde content is observed in Cd-exposed *P. sativum* embryos, which might indicate membrane lipid peroxidation [66]. 

Cadmium is a known inducer of oxidative stress resulting in elevated ROS levels [9,15,67]. Cadmium-induced oxidative stress was able to oxidize Trx isoforms in *P. sativum* seeds [68]. These proteins are potentially involved in monitoring the redox state of storage proteins in both cereals and dicotyledons [69]. Furthermore, the GSH levels were twofold lower, accompanied with a decrease in glutathione reductase (GR) activity, which suggests that intracellular oxidative stress occurred in seeds under Cd exposure [70]. This depletion of the reduced GSH pool might be partially compensated by a higher level of glutaredoxin (Grx) level, which is able to bind Cd at its active site. Peroxiredoxin (Prx) expression was elevated under Cd exposure in both cotyledons and embryonic axes of *P. sativum* seeds. Cadmium could bind to the cysteine residues of Prx, which could serve as a Cd sink [71]. This mechanism might protect the seed from Cd-induced oxidative stress.

Studies that link the interaction between Cd and phytohormones during seed germination are still scarce. Treatment with ethylene is shown to have an alleviating role on Cd-inhibited germination in *Cajanus cajan* [72]. In *O. sativa*, the α-amylase activity is enhanced when Cd-exposed seeds are pre-treated with SA and seedlings show a reduced Cd uptake [73]. In conclusion, seed germination comes forward as a tightly regulated orchestra between hormones and the ROS balance. Within the oxidative window of seed germination hypothesis, one might assume that a small amount of Cd could stimulate the initiation of germination as long as the adverse effects of Cd are not impossible to overcome by the plant’s antioxidant defense mechanisms. 

## 3. Vegetative Plant Growth

**Figure 2:**
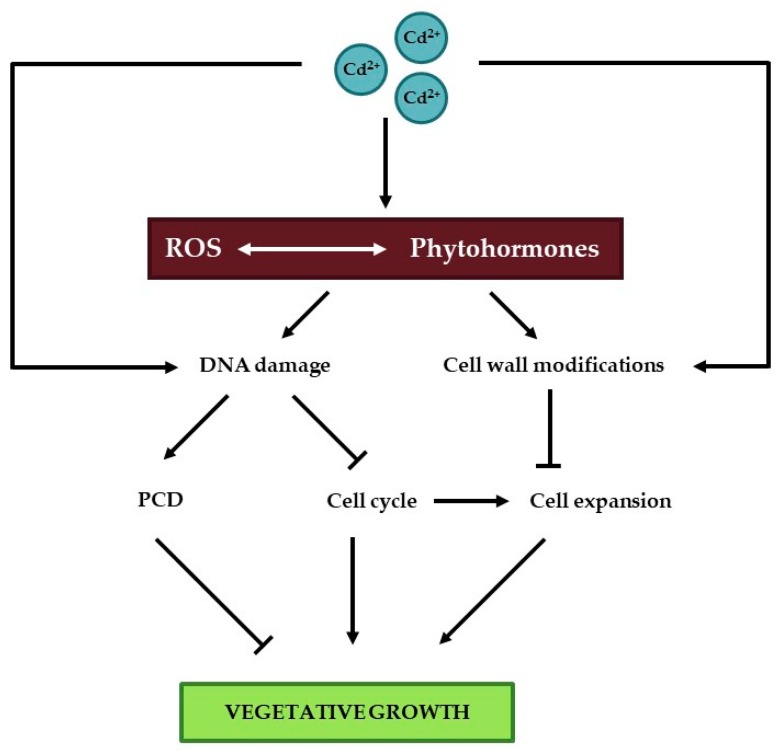
Schematic overview of important players affecting vegetative plant growth upon Cd exposure. Cadmium exposure is well known to affect concentrations of reactive oxygen species (ROS) and phytohormones, which are closely intertwined. Cadmium induces DNA damage, thereby activating the DNA damage response, which can either induce programmed cell death (PCD) or affect cell cycle progression, depending on the extent of DNA damage. In addition, Cd exposure induces cell wall modifications as a strategy to reduce Cd entry into cells. This in turn limits cell expansion, which is intertwined with the cell cycle and specifically endoreduplication. Cadmium-induced DNA damage and cell wall modifications could either result from its effects on ROS and phytohormone levels or arise through an alternative pathway. Together, Cd-induced PCD, cell cycle alterations and inhibition of cell expansion contribute to its negative effect on vegetative growth. One-way arrows indicate stimulating effects, whereas T-shaped arrows represent an inhibitory effect. Two-way arrows signify an interaction.

Once germination has occurred, two major processes drive plant growth, namely cell division and cell expansion, which is limited by the cell wall during vegetative growth. In the following sections, the effect of Cd on these processes is uncovered in detail (Figure 2). 

### 3.1. The DNA Damage Response

Stress-induced effects on cell cycle progression often result from the activation of the DNA damage response (DDR). Upon perceiving DNA damage, cells trigger this response, which includes the activation of DNA repair pathways. When the extent of DNA damage is low, cell cycle progression is transiently inhibited in order to repair the DNA before DNA replication or cell division take place. When the damaged DNA cannot be repaired, cells undergo terminal differentiation or programmed cell death (PCD) [74]. 

The induction of cell cycle arrest upon the perception of DNA damage requires the activation of one of two phosphatidylinositol-3-OH-kinase-like kinases: ataxia telangiectasia mutated (ATM) and ATM- and RAD3-related (ATR). Whereas ATM is mainly activated by the presence of DNA double-strand breaks (DSBs), ATR is involved in responses to stalled replication forks. However, both types of DNA damage often occur simultaneously, causing the activation of both kinases [74]. Whereas cell cycle regulation in response to DNA damage depends on p53 in animals, plants lack a p53 orthologue. Instead, SUPPRESSOR OF GAMMA RESPONSE 1 (SOG1) is considered as the plant counterpart of p53. This transcription factor belongs to the NO APICAL MERISTEM/*ARABIDOPSIS* TRANSCRIPTION ACTIVATION FACTOR/CUP-SHAPED COTYLEDON (NAC) domain family and is activated through phosphorylation by ATM. Once active, SOG1 induces the expression of a multitude of genes involved in DNA repair, cell cycle progression and cell death [74,75]. In addition to SOG1, also WEE1 plays a key role in the plant DDR. This kinase can be activated by both ATM and ATR and mainly controls S phase progression [74]. 

#### 3.1.1. DNA Damage

Cadmium exposure is well known to induce DNA damage in mammalian cells, as reviewed by Bertin and Averbeck (2006) and Filipic (2012) [76,77]. The mechanisms responsible for this Cd-induced DNA damage include ROS-induced formation of 8-hydroxyguanosine and the inhibition of DNA repair systems. Furthermore, Cd is able to interfere with proteins containing a zinc finger motif, implicated in the maintenance of genome stability, DNA repair and DNA damage signaling [76,77].

At present, in-depth knowledge on Cd-induced DNA damage and its underlying mechanisms in plants is lacking. However, many studies have demonstrated that Cd exposure induces DNA damage in multiple plant species. Table 1 provides an overview of recently published research (since 2014) demonstrating different types of Cd-induced DNA damage in a broad range of plant species. Several authors reported that Cd exposure increases the percentage tail length, tail intensity and tail moment determined through single cell gel electrophoresis, also known as the comet assay (Table 1). The detection of DNA damage using this method relies on the fact that DNA strand breaks facilitate DNA migration from the nucleoids towards the anode during gel electrophoresis, thereby forming a “comet tail”. Whereas the alkaline comet assay (performed at pH > 13) detects both DNA single-stranded breaks (SSBs) and DSBs, only DSBs are detected through the neutral comet assay [78,79]. Taken together, the results obtained through this method suggest that Cd exposure induces DNA strand breaks in different plant species including *V. faba* [80,81,82], *Nicotiana tabacum* [83,84], *Allium sativum* [82], *Solanum tuberosum* [85], *Allium cepa* [80,86,87], *Lemna minor* [88,89], *Lactuca sativa* [86,90], *H. vulgare* [91], *Brassica oleracea* and *Trifolium repens* [92]. In *S. tuberosum*, the Cd-induced increase in percentage tail DNA was more pronounced in roots as compared to leaves and appeared later in the latter organ [85]. Similarly, Cd exposure for 24 and 72 h caused significant increases in the percentage tail DNA and the tail moment in *N. tabacum* roots, whereas no effects were observed in leaves [83]. This is likely due to the fact that roots form the entry route for Cd into the plant and are therefore exposed earlier than leaves. Furthermore, roots of both plant species were shown to accumulate higher Cd levels than leaves [83,85]. 

In addition to inducing DNA strand breaks, Cd is also shown to induce micronucleus formation and chromosomal aberrations, which are both detected through microscopic analysis (Table 1). Micronuclei arise when chromosome fragments or entire chromosomes fail to be included in the nuclei of the daughter cells at the end of mitosis, as they do not manage to properly attach to the mitotic spindle during anaphase. They eventually become enclosed by a nuclear membrane and appear as small nuclei after conventional nuclear staining. Micronuclei are generally considered as biomarkers for genotoxicity [93,94]. Chromosomal aberrations frequently detected during mitosis in Cd-exposed plants include anaphase and telophase bridges, sticky chromosomes, chromosome breaks, non-oriented chromosomes and laggards chromosomes [80,86,95,96]]. 

Another approach frequently used to determine Cd-induced genotoxicity in plants is the investigation of random amplified polymorphic DNA (RAPD) profiles. This method consists of a PCR amplification of genomic DNA using multiple short (approximately 10 nucleotides) random primers, followed by gel electrophoresis and visualization of the amplified PCR products. Mutations and other types of DNA damage induced by exposure to stress factors such as Cd can result in the disappearance as well as *de novo* creation of primer annealing sites, thereby yielding an altered RAPD profile [97]. This subsequently allows for calculation of the genomic template stability (GTS) using the following formula: GTS (%) = (1 – a/n) × 100, where “a” represents the number of polymorphic bands in the treated samples and “n” identifies the total number of bands in the control samples [98,99]. As indicated in Table 1, Cd exposure was shown to alter the RAPD profile in both roots and leaves of a broad range of plant species, resulting in a reduced GTS.

Other markers used to assess Cd-induced DNA damage are the full peak coefficient of variation determined via flow cytometric analysis [113] and DNA polymorphisms determined by PCR-based methods (besides RAPD) such as amplified fragment length polymorphism (AFLP), simple sequence repeat (SSR), inter-simple sequence repeat (ISSR) and sequence-related amplified polymorphism (SRAP) [103,104,108,109]]. However, in comparison to the other DNA damage indicators described, the use of these markers in studies evaluating Cd genotoxicity in plants is currently limited.

Despite the large number of studies indicating Cd-induced genotoxicity in plants, its underlying mechanisms are still largely unknown. However, as Cd exposure is well known to induce ROS production, it is likely that DNA oxidation contributes to the observed damage. Therefore, it would be of interest to study the extent of oxidative DNA damage in Cd-exposed plants. Furthermore, it would be interesting to assess the extent of DNA repair in Cd-exposed plants to gain an insight into plant responses to the observed Cd-induced damage. Although methods for the assessment of oxidative DNA damage and DNA repair are widely adopted in animal samples, these methods have so far received little attention in plant research. However, their optimization might significantly enhance our knowledge on DNA damage and repair induced by Cd and other stress factors in plants.

#### 3.1.2. The Cell Cycle

As a consequence of inducing DNA damage, Cd exposure can affect cell cycle progression. The formation of new cells through cell division is the primary driving force for organ growth in plants. Similar to that in other eukaryotes, the classical plant cell cycle consists of four phases: gap 1 (G_1_) phase, DNA synthesis (S) phase, gap 2 (G_2_) phase and mitotic (M) phase [114]. The gap phases enable the control of accurate and full completion of previous phases. As a consequence, many important regulatory mechanisms controlling cell cycle progression operate at the G_1_/S and G_2_/M transitions. During the S phase, nuclear DNA is replicated, whereas the replicated sister chromatids are divided over the two daughter cells arising through cytokinesis during the M phase. The cell cycle is regulated by the activity of cyclin-dependent kinases (CDKs), which are serine/threonine protein kinases that phosphorylate target proteins crucial for cell cycle progression. As their name implies, CDKs form heterodimers with regulatory cyclins in order to become activated [115]. During progression throughout the cell cycle, CDK activity shows a typical pattern, reaching two thresholds: one for DNA replication (S phase) and one for cell division (M phase) [116]. 

In addition to dividing through the classical cell cycle, plant cells can also undergo endoreduplication. During this alternative cell cycle mode, plant cells replicate their nuclear DNA (S phase) without intermittent cell division (M phase), resulting in endopolyploidy (*i.e*., the existence of different ploidy levels in adjacent cells of a species) [117]. 

Similar to the classical cell cycle, endoreduplication is also regulated by the action of CDK-cyclin complexes. During an endocycle, CDK activity only reaches the threshold for DNA replication but not for cell division. Endoreduplication is important for normal plant growth and development, as it is tightly related to cell differentiation and expansion [116]. The importance of endoreduplication in plant development and its connection to cell size become apparent during trichome development. Trichomes are large, single epidermal cells that develop on most aerial parts of *A. thaliana* plants and typically contain three to four branches. These highly specialized structures—involved in plant protection against external stress factors such as herbivory, frost and ultraviolet radiation—require endoreduplication for their development and reach a final DNA content of 32C, with C representing the haploid DNA content [118,119]. Interestingly, trichomes were previously shown to accumulate Cd, possibly to prevent Cd-induced damage at more sensitive sites within the plant [120,121]. In addition to its involvement in plant growth and development, endoreduplication could also serve as a strategy in plant defense against biotic and abiotic stress factors, as reviewed by Scholes and Paige (2015) [122]. Under stress conditions, an increased ploidy level and therefore a higher number of DNA templates could help to sustain genome integrity and stimulate genetic pathways responsible for plant defense [122]. 

Cadmium-induced disturbances of root and leaf growth were shown to coincide with effects on cell division in a wide range of plant species in a multitude of experimental set-ups. An overview of recently published research (since 2014) demonstrating Cd-induced effects on multiple cell cycle-related parameters in several plant species is provided in Table 2. In general, Cd negatively affects cell cycle progression, as becomes apparent from a decreased mitotic index (i.e., the ratio between the number of cells undergoing mitosis and the total cell number), determined via microscopic analysis. As suggested by Monteiro et al. (2012), Cd might bind to the sulfhydryl groups of cysteine residues present in tubulins, thereby affecting microtubule formation and disturbing cell division [90]. Although many studies have addressed the influence of Cd exposure on the cell cycle in roots, knowledge regarding its effects on this process in leaves is scarce. However, Baryla et al. (2001) demonstrated that leaves of Cd-exposed *Brassica napus* plants contained a smaller number of stomatal guard cells and were characterized by a larger mesophyll cell size as compared to their control counterparts, suggesting that Cd also affects the cell cycle in leaves [123]. Similarly, Cd exposure inhibited cell division and increased cell size in both the upper and lower cell layers of young *Elodea canadensis* leaves. Interestingly, this response coincided with a strong Cd-induced disturbance of the typical cell wall structure, which was likely the consequence of the accumulation of large amounts of Cd in the apoplast and the binding of Cd ions to cell wall components [124] (*cfr. infra*). Furthermore, Cd exposure was shown to reduce both adaxial pavement cell number and surface area in different leaves of *A. thaliana* in a time-dependent manner. The decreased cell surface area might be related to a lower extent of endoreduplication in leaves of Cd-exposed plants, as indicated by a significantly decreased endoreduplication factor (i.e., the average number of endocycles that has taken place per cell) [105]. In contrast, other studies reported a decreased percentage of cells with a 2C nuclear DNA content and an increased percentage of cells with a higher nuclear DNA content in roots of *A. thaliana* [101,102] and *P. sativum* [125,126,127]. Although an increased proportion of 4C cells might indicate a cell cycle arrest in G_2_ phase, an increased level of 8C cells points towards an elevated extent of endoreduplication. These data suggest that Cd exposure stimulates this alternative cell cycle variant in roots, whereas inhibiting it in leaves. However, in young *A. thaliana* leaves, an increased extent of endoreduplication was observed shortly after the start of Cd exposure [Hendrix et al., personal communication]. It could be hypothesized that increased ploidy levels confer tolerance to Cd stress, since Talukdar (2014) demonstrated that roots of tetraploid and triploid *Lathyrus sativus* plants were less sensitive to Cd as compared to their diploid counterparts [95]. Furthermore, it is tempting to speculate that the increased extent of endoreduplication is related to a stimulation of trichome development, as these structures are characterized by a high nuclear DNA content [118,119] and provide sites for Cd sequestration [120,121]. However, this hypothesis requires further investigation.

Cadmium-induced effects on the cell cycle in roots and leaves often coincide with alterations in the expression of cell-cycle related genes, such as those encoding cyclins and CDKs (Table 2). Indeed, *CYCB1* expression decreased upon Cd exposure in *Glycine max* cell suspension cultures, possibly affecting G_2_/M progression [128,129]. Exposure to 100 µM Cd for 15 days affected the expression of a large number of cell cycle-related genes in roots of *O. sativa*. Interestingly, Cd-induced effects on a number of these genes were altered by simultaneous treatment with 2,3,5-triiodobenzoic acid (an inhibitor of polar AUX transport), indole-3-butytric acid (an AUX hormone), Tiron (a superoxide (O_2_^•−^) scavenger) or sodium diethyldithiocarbamate (an SOD inhibitor) [130]. In another study, the authors demonstrated that treatment with ABA or tungstate (an ABA inhibitor) also influenced Cd-induced effects on transcript levels of certain genes involved in cell cycle regulation [131]. These data emphasize the involvement of plant hormones and ROS in Cd-induced effects on the cell cycle. The significance of various phytohormones and their complex interactions in regulating cell division and endoreduplication was extensively reviewed by Tank et al. (2014) [132]. Although the precise role of ROS in cell cycle regulation is still unclear, their importance in this process is illustrated by the fact that oxidative and reductive signals are required for certain cell cycle transitions. In addition, transcript levels and activities of cyclins and CDKs were demonstrated to be altered upon redox perturbations. The antioxidative metabolite GSH might constitute an important redox-related cell cycle regulator, as it was reported to be translocated into the nucleus during cell division [133] and the severely GSH-deficient *root meristemless 1* (*rml1*) *A. thaliana* mutant is unable to form a root apical meristem due to a lack of cell division [134]. Furthermore, ROS and redox homeostasis are also crucial mediators of cytokinesis, as indicated by a disturbed cell division in mutants characterized by impaired ROS production or signal transduction [135,136]. The involvement of ROS and redox regulators in the cell cycle is discussed in more detail in a recent review by Mhamdi and Van Breusegem (2018) [137].

Additional evidence for Cd-induced effects on transcript levels of cell cycle-related genes comes from *A. thaliana*. In roots of this species, Cd exposure caused a downregulation of several G_1_/S marker genes such as *HISTONE H4* and *E2Fa* and G_2_/M marker genes, including *CYCB1;1* and *CYCB1;2*. This response coincided with an altered expression of DNA repair genes and was less pronounced in mutants with an impaired DNA mismatch repair pathway, suggesting that DNA damage contributes to the observed cell cycle arrest [101,102]. In agreement with this hypothesis, Hendrix et al. (2018) demonstrated significant increases in the expression of several DNA repair genes and genes encoding CDK inhibitors of the SIAMESE-related (SMR) family in leaves of *A. thaliana* exposed to 5 µM Cd for 72 h [105]. Interestingly, these *SMR* genes were previously reported to be transcriptionally upregulated in response to ROS-induced DNA damage in plants exposed to hydroxyurea [138], further pointing towards the involvement of DNA damage in the Cd-induced cell cycle inhibition. 

#### 3.1.3. Cell Death

In case DNA damage is severe and cannot be repaired, the DDR is responsible for activating PCD [74]. Plant PCD is defined as a genetically encoded and actively controlled form of cellular suicide and is generally subdivided into developmental PCD and environmentally induced PCD. Developmental PCD plays a crucial role in normal plant growth and development, being involved in seed development and germination, as well as vegetative development. During the latter, PCD is for example involved in the differentiation of xylem tracheary elements, the emergence of lateral and adventitious roots and in senescence [140]. It is well-known that ROS and NO are important regulators of both developmental and environmentally induced plant PCD, as was recently reviewed by Locato et al. (2016) [140].

Interestingly, Cd-induced DNA damage and cell cycle effects observed in different plant species often coincide with a decrease in cell viability. For example, Kuthanova et al. (2008) demonstrated that exposure to 50 µM Cd affected cell cycle progression in synchronized tobacco BY-2 cell cultures and significantly reduced cell viability [141]. The type of cell death induced upon Cd exposure strongly depends on the cell cycle phase at which Cd exposure started. Whereas Cd application during S and G_2_ phase resulted in an apoptosis-like PCD type, characterized by DNA fragmentation, no such effect was observed in cells exposed to Cd during the other cell cycle phases. Instead, cells exposed during M phase displayed a rapid cell death, coinciding with fragmented late telophasic nuclei, while the decrease in viability of cells treated with Cd at G_1_ phase took place at a slower rate [141]. Similarly, the decreased mitotic index and chromosomal aberrations observed in *A. cepa* root tips upon exposure to a range of Cd concentrations was accompanied by a strong increase in Evans blue staining, indicative of cell death [80]. However, no effect on cell viability was observed in root tips of *T. aestivum* exposed to a range of Cd concentrations for 48 h, although these conditions induced oxidative modifications of cell cycle-related proteins and cell cycle arrest [142]. These data emphasize that the occurrence of Cd-induced cell death depends on many factors including the plant species, Cd concentration and exposure duration.

The Cd-induced PCD response in tobacco BY-2 was shown to depend on three ROS waves: (1) an initial transient NADPH oxidase-dependent increase in H_2_O_2_ levels, (2) the accumulation of mitochondrial O_2_^•−^ and (3) fatty acid hydroperoxide accumulation, which coincides with the occurrence of cell death [143]. The importance of ROS in Cd-induced PCD is also supported by the work from Tamás et al. (2017) [144], who showed that cell death in roots of Cd-exposed *H. vulgare* is mostly pronounced at locations where O_2_^•−^ generation was observed (i.e., the transition and distal elongation zones). However, Cd-induced cell death was only observed in roots of plants exposed to 60 µM Cd, whereas no effect was observed upon exposure to lower concentrations, suggesting that cell death only occurs when ROS concentrations exceed a certain threshold [144]. In an *Arabidopsis* cell suspension culture, the H_2_O_2_ production required for PCD was shown to depend on NO production, as inhibition of NO synthesis partially prevented H_2_O_2_ accumulation and cell death [145]. As summarized by Locato et al. (2016) [140], different underlying mechanisms have been proposed for the observed NO-dependency of Cd-induced PCD. First, NO-dependent nitrosylation of PCs could reduce their ability to chelate Cd, thereby increasing free Cd levels and enhancing toxicity symptoms [145,146]. Furthermore, NO could increase Cd accumulation by affecting transcript levels of genes involved in Cd uptake and detoxification [147,148]. A final mechanism proposed is the Cd-induced activation of MPK6, which subsequently activates caspase-3-like, a PCD executor [149].

It is likely that phytohormones are also involved in regulating Cd-induced PCD, possibly through interactions with ROS. Simultaneous exposure of a tomato cell suspension culture to Cd and ethylene caused a stronger Cd-induced PCD response as compared to treatment with Cd alone. Furthermore, Cd-induced PCD was mitigated by application of the ethylene biosynthesis inhibitor 2-aminoethoxyvinyl glycine and the ethylene receptor blocker silver thiosulfate [150], clearly indicating the key role of ethylene signaling in Cd-induced PCD. Furthermore, a Cd-induced increase in cellular SA concentrations of tobacco cells was shown to induce a MAPK signaling pathway involved in mediating PCD [151]. Similarly, exogenously applied SA was able to alleviate Cd-induced ROS production, photosynthetic damage and cell death in Cd-exposed *A. thaliana* [152]. The cell death observed in this study was likely a consequence of a strong Cd-induced activation of autophagy. This process involves the vacuolar degradation and recycling of cellular macromolecules or entire organelles and was previously reported to be induced upon Cd exposure in various plant species including *A. thaliana* [152], *G. max* [153], *T. aestivum* [154] and *Theobroma cacao* [155]. Although their interplay has not yet been studied in Cd-exposed plants, crosstalk between autophagy and several phytohormones exists in plants exposed to other environmental stimuli or during normal plant development [156]. Furthermore, an interplay between autophagy and ROS was also shown, with ROS contributing to the establishment of autophagy and autophagy contributing to ROS scavenging. A comprehensive overview of the involvement of ROS and phytohormones and their interplay in autophagy in stress-exposed plants was recently published by Signorelli et al. (2019) [157]. However, the involvement and interplay of ROS and phytohormones in Cd-induced autophagy remains largely unknown. Interestingly, autophagy contributes to nutrient recycling and remobilization during senescence [157], a process which was shown to be prematurely induced upon Cd exposure in different plant species, as indicated by increases in senescence-related parameters such as protease activity, lipid peroxidation and the expression of senescence-associated genes (SAGs) [9].

Taken together, these data suggest that Cd-induced DNA damage is an important trigger for effects on plant growth either through the induction of cell death or through influences on the cell cycle. In order to prevent Cd entry into cells and subsequent DNA damage, cell wall structure can be altered to increase the number of Cd binding sites. This often coincides with an increased cell wall rigidity, which can in turn limit cell expansion and hence plant growth.

### 3.2. The Cell Wall 

The primary cell wall consists of structural proteins that are embedded in a matrix of polysaccharides which includes cellulose, hemicellulose and pectin [158,159,160]. These components ensure that inner structures have sufficient support, but at the same time remain adjustable for the expanding cell. Furthermore, these polysaccharides contain functional groups such as hydroxyl, thiol and carboxyl groups that enable the cell wall to bind large amounts of divalent and trivalent metals including Cd [161]. The homogalacturonan (HG) domain of pectin is created by the golgi apparatus and secreted into the cell wall in a highly methylesterified form. Using a Ca ion, two HG domains can be linked together by their carboxylic groups [162]. Calcium can be replaced within this structure by other metals such as Pb, Cu, Cd and Zn that show greater affinity [161].

#### 3.2.1. The Cell Wall as Major Storage Compartment for Cadmium 

The cell wall is the primary defense structure of a plant’s cell against pathogen attacks or unfavorable environmental conditions such as drought and metals [163,164,165]. By keeping excess Cd out of the cytoplasm, it prevents damage to macromolecules, proteins and DNA caused directly or upon Cd-induced oxidative stress. Since roots are the organs in direct contact with Cd from the soil, their cell walls play an important role in this process. Many studies show that most Cd is stored within the cell walls of roots by various plant species [166,167]. For example, in the plant *Coptis chinensis*, the roots and rhizomes retained between 62 and 77% of all Cd [168]. When the capacity of the cell wall is exceeded, Cd might additionally form complexes with PCs and is subsequently sequestered within the vacuole [167,169,170]. However, Dong and colleagues (2016) reported that upon exposure to 200 µM CdCl_2_, most Cd in the leaves was present in the soluble fraction in *Arachis hypogaea* and compartmentalization in vacuoles might be of greater importance here [171].

#### 3.2.2. Cadmium-Induced Cell Wall Modifications 

In addition to the formation of a rigid barrier, the cell wall might also be actively modified under Cd exposure [172,173,174]. By increasing the pectin methylesterase (PME) activity, the amount of de-esterified pectin increases, thereby creating more negative charges to bind Cd [175,176]. Moreover, an increase in pectin and hemicellulose content can also contribute to the latter [177,178]. Recently, in *O. sativa* seedlings it was shown that root aeration increases pectin content and PME activity resulting in Cd toxicity alleviation [179]. Since pectin is especially synthesized in young, expanding cells, a delay in root maturation due to aeration might enhance pectin deposit. Next to cell wall modifications related to primary components, another effect that occurs under Cd exposure, is the activation of lignin biosynthesis [180,181]. Lignin is incorporated in the secondary cell walls of specialized cells such as tracheids and vessel elements of the xylem and is created by the oxidative polymerization of monolignol subunits by class III peroxidases (PODs) and laccases [182]. Adding lignin to the cells’ walls decreases the permeability to Cd, but at the same time restricts cell elongation, thereby inhibiting growth. An increase of POD activity in response to Cd has been observed in various plant species [180,181,183]. It is of particular interest that POD uses H_2_O_2_ as a substrate, but at the same time H_2_O_2_ comes forward as a major signaling molecule within the redox network, which is strongly disturbed by Cd exposure. These close interactions between lignification and redox regulation have been extensively reviewed by Loix and colleagues (2017) [173]. Cell wall expansion is largely regulated by ROS homeostasis in the apoplast [184]. Whereas H_2_O_2_ contributes to cell wall stiffening, hydroxyl radicals (^•^OH) lead to cell wall loosening due to pectin and xyloglucan cleavage [185,186]. 

The role of cellulose under Cd exposure is still a matter of debate. The cell wall cellulose content was shown to decrease in *O. sativa* and *Zea mays* under Cd exposure, but an increase was observed in *Linum usitatissimum*. Therefore, it is proposed that different defense strategies are present between monocots and dicots [175,177,187]. Recently, it was shown that Cd accumulates preferably within cellulose in the Cd-sensitive *G. max* BX10 variety, but contrarily, the Cd-tolerant *G. max* HX3 accumulated more Cd in cell wall-related pectin [188]. This might even suggest intraspecies differences in sequestration of Cd in different cell wall components. Cellulose microfibrils are cable-like structures composed of many β-1,4-linked glucose molecules, which are added together by cellulose synthase using uridine diphosphate glucose as a donor molecule [189]. Cadmium is known to reduce the amount of cytosolic sucrose, which could have detrimental effects on cellulose biosynthesis, as UDP-glucose is thought to be mainly produced by sucrose synthase (SUSY) from sucrose [174,190]. In a recent proteomic study by Gutsch and colleagues (2018), SUSY was shown to be upregulated in response to long-term Cd exposure in *Medicago sativa* [191]. The photosynthetic capacity of these plants was inhibited, as indicated by a lower abundance of photosynthetic proteins, which cuts off glucose supply for cell wall biosynthesis. However, a higher SUSY activity might suggest that the plants still invested in cellulose synthesis. In *Miscanthus sacchariflorus*, several genes involved in cellulose biosynthesis were upregulated under Cd exposure as well. These included cellulose synthase A, cellulose synthase-like protein D4 and cellulose synthase-like protein H1 [192].

In addition to these polysaccharides and lignin, two protein families, i.e., xyloglucan endotransglucosylases/hydrolases (XTH) and expansins (EXP), were demonstrated to affect cell wall expansion as well [193]. Expansins have a function in breaking the non-covalent binding of polysaccharides resulting in cell wall loosening [158,194]. In *N. tabacum*, *NtEXP1*, *NtEXP4* and *NtEXP5* transcripts were abundant in the shoot apices and young leaves, but not in roots and mature leaves, supporting their function in cell wall extension [195]. Furthermore, these genes were induced by growth-stimulating hormones such as AUX, GA and CK, but Cd exposure inhibited their transcription. This was supported by a study in *Brassica juncea* by Sun and colleagues (2011) who found that overexpression of *BjEXPA1* led to a higher Cd sensitivity [196]. However, the opposite was found for the *TaEXP2* gene in *T. aestivum* [197]. This gene was upregulated under Cd exposure and its overexpression led to an increase in biomass and root elongation. This improved plant performance was stated to be due to an enhanced translocation of Cd to the vacuoles, a higher antioxidant capacity and a higher water retention. Secondly, XTHs have the ability to cut and rejoin xyloglucan, which locks cellulose microfibrils and thereby contributes to cell wall loosening [198]. In *A. thaliana*, *XTH33* was required for Cd accumulation within the roots [199]. In addition, *XTH33* was shown to be a direct target of EIN3 which acts as a master transcription factor in ethylene-mediated Cd-induced root growth inhibition. When *PeXTH* from *Populus euphratica* was introduced in *N. tabacum*, a decrease of xyloglucan was observed in the cell wall of the roots, leading to a reduced number of Cd binding sites, thereby reducing Cd influx into the roots and limiting Cd toxicity [200]. Lastly, in a proteomic study with two cultivars of *A. hypogaea*, it was hypothesized that XTHs and α-expansins might be important in keeping cell wall extensibility under Cd exposure in the Cd-sensitive cultivar, which showed a higher capacity of cell wall modification [201]. Both XTH and EXP proteins are activated by AUX, inducing acidic growth of the cell wall [202]. 

#### 3.2.3. The Role of The Cell Wall in Cadmium Hyperaccumulators 

Plants that are able to withstand very high metal concentrations in the soil and show an enhanced metal accumulation within the aboveground organs are defined as hyperaccumulators [169]. These remarkable plant species account for less than 0.2% of all angiosperms, although additional heavy metal-accumulating plants are likely to be identified [203]. The trait is expected to have evolved multiple times but is of very high occurrence within the Brassicaceae family [169]. It is a nice coincidence that frequently studied hyperaccumulators such as *Arabidopsis halleri* and *Noccea caerulescens*, previously known as *Thlaspi caerulescens*, are phylogenetic closely related to the model plant *A. thaliana*, which does not accumulate metals. As shown by Benzarti et al. (2008), the Cd hyperacummulator *N. caerulescens* has a more than tenfold higher EC50 value for Cd-induced root growth inhibition in comparison to several non-accumulator plants [204]. However, large differences in the ability to accumulate Cd also exist between populations of hyperaccumulating species [203]. In *A. halleri*, some individuals of metallicolous populations were able to survive in the presence of 450 µM CdSO_4_, whereas concentrations as low as 100 µM CdSO_4_ caused mortality within non-metallicolous populations [205]. Tolerance mechanisms to cope with high soil metal concentrations include enhanced metal uptake and xylem loading, followed by detoxification in the shoot, as reviewed by Verbruggen and colleagues (2009) [206]. 

The role of Cd storage in the cell wall of hyperaccumulators might not be that straightforward. In a comparative study between a non-hyperaccumulating and a hyperaccumulating ecotype of *Sedum alfredii*, it was shown that for the latter more cell wall-bound Cd was available for xylem loading [207]. Furthermore, no increase of pectin or hemicellulose 2 was detected in the hyperaccumulating ecotype, which was accompanied with a lower PME activity. This led the authors to the conclusion that Cd translocation could at least partly be due to a difference in cell wall modification regulation [207]. In addition, cell wall modifications in the shoot cell walls of *A. halleri* under Cd exposure were more pronounced in less tolerant populations [205]. A Cd tolerance mechanism might already be present under controlled conditions in *A. halleri* in contrast to *A. thaliana* [208]. Gene expression related to redox balance, Ca signaling, and cell wall remodeling was more affected in *A. thaliana*, yet *PME* and *CESA* transcripts (encoding for cellulose synthases) were more upregulated in *A. halleri*. This could result in a larger extent of Cd binding to the cell wall and cell wall stiffening, which in turn leads to a greater barrier for cytosolic entering [208]. Moreover, Peng and colleagues (2017) showed that the cell wall of a newly discovered hyperaccumulator *Sedum plumbizincicola* plays a crucial role in Cd tolerance [209]. In contrast to a non-hyperaccumulating ecotype of *S. alfredii*, Fourier transform infrared (FT-IR) spectroscopy displayed a higher absorbance ratio of –COO^−^ against –COOR, indicating a lower esterification of pectin and a more efficient binding of metal ions for *S. plumbizincicola*. 

## 4. Reproductive Growth 

In order for a plant to complete its life cycle, it must commence the reproductive phase, which in higher plants involves the setting of flowers, pollination and fertilization, followed by the production of seeds. In *Brassica campestris*, the GSH and AsA content dropped the most when plants were exposed to Cd at their flowering stage, indicating that the reproductive phase is highly susceptible [210]. In *L. usitatissimum* plants grown in Cd-contaminated soils, a decrease in fitness was noted due to 31.8% fewer seeds and 25.6% fewer fruits [211].

Although *A. thaliana* plants showed a reduction in silique counts under long-term Cd exposure, they were still able to complete their life cycle, thereby producing seeds with equal germination capacity as control plants [212]. The transition from the vegetative to the reproductive phase under 10 µM Cd was unaffected, which was in agreement with the results from Maistri and colleagues (2011), however an accelerated emergence of inflorescence was observed under 5 µM Cd [213]. Limited research has been done on the later stages of plant development in relation to Cd exposure. Nevertheless, accumulation of large amounts of Cd in the seeds, even without visible toxic effects to the plant, can be a great threat to human health [214]. Cadmium uptake by roots and translocation to aboveground parts is a process that continues throughout the plant’s life cycle. In *O. sativa*, most of the Cd accumulated in the grains during the early phases of grain development either directly via the xylem or through remobilization through the phloem [215]. However, in *Solanum lycopersicum* the majority of Cd was taken up in the final stage of fruit development [216]. This co-occurred with a disturbance in nutritional status, as potassium (K), Fe and Zn contents in fruits decreased, while Ca and magnesium (Mg) increased. Exposure to 100 µM Cd resulted in an absence of fruit setting. Furthermore, an interesting finding was reported in the monoecious plant *Crocus sativus*, where a shift to more male flowers was apparent upon Cd exposure [217]. This was also observed in a study with *Cannabis sativa*, where treatment with Pb resulted likewise in male flowers and was demonstrated to be due to a hormonal shift with increasing GA levels in these plants, while zeatine (a specific form of CK) decreased [218]. 

Cadmium has been shown to negatively affect pollen germination accompanied with a disruption of pollen tube morphology in multiple plant species [219,220]. Even very low Cd concentrations of 0.01 µg mL^−1^ were able to inhibit either pollen germination or tube growth in *Vicia angustifolia* and *Vicia tetrasperma*, indicating that this process is very sensitive to Cd [221]. The impaired cell elongation of the pollen tube by Cd is a consequence of its interference with the anionic content of secretory vesicles and its interaction with the cell wall, which contains large quantities of pectin and callose [222]. Cell wall thickening, an increase in cell diameter and abnormal pollen tube growth were observed in all *Prunus avium* cultivars tested in vitro under Cd exposure [223]. Pollen tubes of *Picea wilsonii* showed swelling of the tips accompanied by cytoplasmic vacuolization [224]. Furthermore, the importance of ROS/Ca signaling in pollen tube formation has been well documented [137,225]. Exogenously applied ^•^OH to pollen of *N. tabacum* caused loosening of the intine (i.e., the inner layer of the pollen tube cell wall), leading to a disrupted polar growth, while H_2_O_2_ stiffened the cell wall [226]. Both treatments resulted in a reduced pollen germination, since only the region of the germinating pore should be weakened through ^•^OH-dependent reactions. In conclusion, Cd negatively affects plant fitness by interfering with various processes including pollen tube formation and pollen germination resulting in smaller numbers of seeds that show a reduced germination as was described in the section on seed germination. In conclusion, knowledge on the effects of Cd on the reproductive phase of plant development is still relatively scarce in contrast to information on its effects on vegetative growth and deserves more attention in the future.

## 5. Conclusion and Future Perspectives

Cadmium exposure interferes with all stages of plant development, inhibiting seed germination, vegetative growth and reproductive growth. Important players in the Cd-induced disturbance of root and leaf growth are DNA damage, which subsequently affects cell cycle progression or even causes cell death, and structural alterations of the cell wall. Furthermore, Cd alters pollen tube morphology, inhibits pollen germination and can be transported into seeds and fruits. The type and extent of Cd-induced effects strongly depends on many factors, including the plant species, organ, cell type, Cd concentration and exposure duration. 

Additional research on the mechanisms underlying these Cd-induced disturbances of plant growth and development is required to develop and further improve strategies to enhance plant growth on Cd-polluted soils. In this context, it would be interesting to also focus on transgenerational effects involved in plant adaptation to Cd exposure. Although research in this field is currently limited, a recent study showed that Cd-induced alterations of RAPD profiles in *Urtica pilulifera* parent plants are transmitted to the next generation [112]. Similarly, Carvalho et al. (2018) demonstrated that exposure of *S. lycopersicum* to Cd resulted in Cd accumulation in seeds, which altered their nutrient profile and caused a decreased mitotic index in root tips of the offspring [227]. Although the mechanisms underlying these transgenerational effects are currently unknown, epigenetic changes might be involved, as Cd exposure was previously shown to alter DNA methylation patterns in *A. thaliana* [104]. In addition, the possible involvement of the microbiome in plant adaptation to Cd exposure should also be taken into account, as transgenerational exposure of *A. thaliana* to Cd significantly altered the seed endophytic community [228,229]. Furthermore, inoculation with specific plant-associated bacteria improved root growth of Cd-exposed *A. thaliana* plants [230], emphasizing the importance of the plant microbiome in coping with environmental stress factors and optimizing plant growth and development under suboptimal conditions. Finally, the use of soil amendments such as biochar and silicon-based fertilizers is promising for future applications to alleviate Cd toxicity, thereby improving crop growth and quality [231,232].

## Figures and Tables

**Table 1:** Overview of recent research articles (published since 2014) demonstrating Cd-induced DNA damage, arranged by plant species. Cadmium is shown to induce different types of DNA damage, including DNA strand breaks, chromosomal aberrations and micronuclei in different plant species. Furthermore, it alters the expression of DNA repair genes and changes amplified fragment length polymorphism (AFLP), inter-simple sequence repeat (ISSR), random amplified polymorphic DNA (RAPD), sequence-related amplified polymorphism (SRAP) and simple sequence repeat (SSR) profiles, thereby reducing the genomic template stability (GTS). ↑ and ↓ symbols indicate increases and decreases, respectively.

Species	Organ	Cd Concentration	Exposure Duration	Effect	Detection Method	Reference
*Allium cepa*	Root tip	50–200 µM	2 h + 24 h recovery	Micronucleus formation	Microscopic analysis	Arya and Mukherjee, 2014 [80]
				Chromosomal aberrations		
				% tail DNA ↑	Comet assay (alkaline)	
	Root tip	25 µM	48 h	Chromosomal aberrations	Microscopic analysis	Silveira et al., 2017 [86]
				Micronucleus formation		
				% DNA damage ↑	Comet assay (alkaline)	
	Root tip	25 µM	48 h	Number of nucleoli ↑	Microscopic analysis	Lima et al., 2019 [100]
*Arabidopsis thaliana*	Root tip	0.125–2.5 mg L^−1^	5 d	Altered expression DNA repair genes	qRT-PCR	Cui et al., 2017 [101]
	Root	1.25–4 mg L^−1^	5 d	Altered RAPD profile	RAPD	Cao et al., 2018 [102]
				Altered expression DNA repair genes	qRT-PCR	
	Leaf	0.5–5 mg L^−1^	16 d	Altered AFLP profile	AFLP	Li et al., 2015 [103]
	Leaf	0.25–8 mg L^−1^	15 d	Microsatellite instability	SSR	Wang et al., 2016 [104]
				Altered RAPD profile	RAPD	
	Leaf	5 µM	72 h	Altered expression DNA repair genes	qRT-PCR	Hendrix et al., 2018 [105]
*Brassica chinensis*	Leaf	15–120 mg kg^−1^ soil	30 d	Altered RAPD profile	RAPD	Sudmoon et al., 2015 [106]
*Brassica oleracea*	Root	2.5–20 mg kg^−1^ soil	3–56 d	Altered % tail intensity	Comet assay (alkaline)	Lanier et al., 2019 [92]
*Capsicum annuum*	Root tip	20–100 ppm	24 h	Chromosomal aberrations	Microscopic analysis	Aslam et al., 2014 [96]
	Leaf	20–100 ppm	24 h	Altered RAPD profile	RAPD	
*Hordeum vulgare*	Root tip	75–225 µM	7 d	Altered RAPD profile (GTS ↓)	RAPD	Cenkci and Dogan, 2015 [98]
	Leaf	5 µM	15 d	DNA damage ↑	Comet assay (alkaline)	Cao et al., 2014 [91]
*Ipomoea aquatica*	Entire seedling	15–120 mg kg^−1^ soil	21 d	Altered RAPD profile (GTS ↓)	RAPD	Tanee et al., 2016 [99]
*Lactuca sativa*	Root tip	25 µM	48 h	Chromosomal aberrations	Microscopic analysis	Silveira et al., 2017 [86]
				Micronucleus formation		
				% DNA damage ↑	Comet assay (alkaline)	
*Lathyrus sativus*	Root tip	5–50 µM	3–7 d	Chromosomal aberrations	Microscopic analysis	Talukdar, 2014 [95]
				Micronucleus formation		
*Leucaena leucocephala*	Leaf	50 mg L^−1^	15 d	Altered RAPD profile	RAPD	Venkatachalam et al., 2017 [107]
*Nicotiana tabacum*	Root and leaf	10–15 µM	7 d	% tail DNA ↑	Comet assay (alkaline)	Tkalec et al., 2014 [84]
*Oryza sativa*	Root tip	50–200 µM	48–96 h	Altered SRAP profil (GTS ↓)	SRAP	Zhang et al., 2015 [108]
*Sphagnum palustre*	Shoot	0.1–10 µM	24–48 h	Altered ISSR profile (GTS ↓)	ISSR	Sorrentino et al., 2017 [109]
*Trifolium repens*	Root	2.5–20 mg kg^−1^ soil	3–56 d	Altered % tail intensity	Comet assay (alkaline)	Lanier et al., 2019 [92]
	Root and leaf	20–60 mg kg^−1^ soil	2 weeks	Altered RAPD profile	RAPD	Ghiani et al., 2014 [110]
	Leaf	2.5–20 mg kg^−1^ soil	3–56 d	Tail moment ↑	Comet assay (alkaline)	Lanier et al., 2016 [111]
*Urtica pilulifera*	Root tip	100–200 µM	2 months	Altered RAPD profile	RAPD	Dogan et al., 2016 [112]
*Vicia faba*	Root tip	50–200 µM	2 h + 24 h recovery	Micronucleus formation	Microscopic analysis	Arya and Mukherjee, 2014 [80]
				Chromosomal aberrations		
				% tail DNA ↑	Comet assay (alkaline)	

**Table 2:** Overview of recent research articles (published since 2014) demonstrating Cd-induced effects on cell cycle-related parameters, arranged by plant species. Cadmium exposure is shown to reduce the mitotic index (i.e., the ratio between the number of cells undergoing mitosis and the total cell number), alter nuclear ploidy levels and affect the expression of cell cycle-related genes in different plant species. ↑ and ↓ symbols indicate increases and decreases, respectively. EdU: 5-ethynyl-2′-deoxyuridine; FCM: flow cytometry; qRT-PCR: quantitative reverse transcription polymerase chain reaction; RT-PCR: reverse transcription polymerase chain reaction.

Species	Organ	Cd Concentration	Exposure Duration	Effect	Detection Method	Reference
*Allium cepa*	Root tip	50–200 µM	2 h + 24 h recovery	Mitotic index ↓	Microscopic analysis	Arya and Mukherjee, 2014 [80]
	Root tip	25 µM	48 h	Mitotic index ↓	Microscopic analysis	Silveira et al., 2017 [86]
*Arabidopsis thaliana*	Root tip	0.125–2.5 mg L^−1^	5 d	2C ↓, 4C ↑, 8C ↑	FCM	Cui et al., 2017 [101]
				Altered cell cycle phase distribution		
				Altered expression cell cycle-related genes	qRT-PCR	
	Root	1.25–4 mg L^−1^	5 d	2C ↓, 4C ↑	FCM	Cao et al., 2018 [102]
				Altered expression of cell cycle-related genes	qRT-PCR	
	Leaf	5 µM	3–12 d	Endoreduplication factor ↓	FCM	Hendrix et al., 2018 [105]
				Epidermal cell number and cell surface area ↓	Microscopic analysis	
				Altered expression of cell-cycle related genes	qRT-PCR	
*Capsicum annuum*	Root tip	20–100 ppm	24 h	Mitotic index ↓	Microscopic analysis	Aslam et al., 2014 [96]
*Lactuca sativa*	Root tip	25 µM	48 h	Mitotic index ↓	Microscopic analysis	Silveira et al., 2017 [86]
*Lathyrus sativus*	Root tip	5–50 µM	3–7 d	Mitotic index ↓	Microscopic analysis	Talukdar, 2014 [95]
*Oryza sativa*	Root	200 µM	7 d	Cortex cell length in elongation zone ↓	Microscopic analysis	Zhao et al., 2014 [131]
				Cortex cell number in elongation zone ↓		
			7–11 d	Altered expression of cell cycle-related genes	RT-PCR	
*Sorghum bicolor*	Root tip	50–200 µM	5 d	Inhibition of S phase progression	EdU assay	Zhan et al., 2017 [139]

## Abbreviations

ABA	Abscisic acid
ABI3	Abscisic acid insensitive 3
ACC	1-aminocyclopropane-1-carboxylate
ACS	ACC synthase
AFLP	Amplified fragment length polymorphism
AsA	Ascorbate
AUX	Auxin
ATM	Ataxia telangiectasia mutated
ATR	RAD3-related
Ca	Calcium
CAT	Catalase
Cd	Cadmium
CDKs	Cyclin-dependent kinases
Cr	Chromium
CK	Cytokinin
DDR	DNA damage response
DSBs	Double-stranded breaks
EdU	5-ethynyl-2′-deoxyuridine
ET	Ethylene
EXP	Expansin
FCM	Flow cytometry
Fe	Iron
FT-IR	Fourier transform infrared
GA	Gibberellic acid
GR	Glutathione reductase
Grx	Glutaredoxin
GSH	Glutathione
GTS	Genomic template stability
H_2_O_2_	Hydrogen peroxide
HG	Homogalacturonan
IAA	Indole-3-acetic acid
ISSR	Inter-simple sequence repeat
JA	Jasmonic acid
Mn	Manganese
NAC	NO APICAL MERISTEM/ARABIDOPSIS TRANSCRIPTION ACTIVATION FACTOR/CUP-SHAPED COTYLEDON
NADPH	Nicotinamide adenine dinucleotide phosphate
^•^OH	Hydroxyl radical
O_2_^•^	Superoxide
Pb	Lead
PC	Phytochelatin
PCD	Programmed cell death
PME	Pectin methylesterase
POD	Class III peroxidase
Prx	Peroxiredoxin
qRT-PCR	Quantitative reverse transcription polymerase chain reaction
RAPD	Random amplified polymorphic DNA
ROS	Reactive oxygen species
RT-PCR	Reverse transcription polymerase chain reaction
SA	Salicylic acid
SAGs	Senescence-associated genes
SMR	SIAMESE-related
SOD	Superoxide dismutase
SOG1	SUPPRESSOR OF GAMMA RESPONSE 1
SRAP	Sequence-related amplified polymorphism
SSBs	Single-stranded breaks
SSR	Simple sequence repeat
SUSY	Sucrose synthase
TFs	Transcription factors
Trx	Thioredoxin
XTH	Endotransglucosylases/hydrolases
Zn	Zinc
